# The relationship between socioeconomic position and health literacy among urban and rural adults in regional China

**DOI:** 10.1186/s12889-021-10600-7

**Published:** 2021-03-17

**Authors:** Wei Chen, Hongfu Ren, Na Wang, Yaqing Xiong, Fei Xu

**Affiliations:** 1 Nanjing Gulou District Center for Disease Control and Prevention, Nanjing, China; 2 Department of Epidemiology, School of Public Health, Nanjing Medical University, 101 Longmian Ave., Nanjing, 211166 People’s Republic of China; 3 Nanjing Municipal Center for Disease Control and Prevention, Nanjing, China; 4 Geriatric Hospital of Nanjing Medical University, 30 Luojia Rd., Nanjing, 210024 People’s Republic of China

**Keywords:** Health literacy, Socioeconomic position, Education, Family average income, Occupation

## Abstract

**Background:**

To examine associations of socioeconomic position (SEP), separately indicated by education, monthly family average income (FAI) and occupation, with health literacy (HL) among adults in regional China.

**Methods:**

A cross-sectional survey was conducted among urban and rural adults (aged 25–69 years) who were randomly selected, using the probability proportionate to size sampling approach, from Nanjing municipality of China during October and December of 2016. HL, the outcome variable, was assessed using the Chinese Resident Health Literacy Scale. SEP, our independent variable, was separately measured with educational attainment, monthly family average income and occupation. Logistic regression models were introduced to examine SEP-HL association with odds ratio (OR) and 95% confidence interval (CI).

**Results:**

Totally, 8698 participants completed the survey. The proportion of participants with unweighted and weighted adequate HL was 18.0% (95%CI = 17.2, 18.8%) and 19.9% (95%CI = 16.6, 23.6%), respectively, in this study. After adjustment for possible confounding factors, each SEP indicator was in significantly positive relation to both unweighted and weight HL level. Participants who obtained 13+ and 10–12 years educational attainment, respectively, had 2.41 (95%CI = 1.60, 3.64) and 1.68 (95%CI = 1.23, 2.29) times odds to record weighted adequate HL compared to their counterparts who were with 0–9 years education. Subjects within upper (OR = 1.92, 95%CI = 1.24, 2.98) and middle FAI tertile (OR = 1.59, 95%CI = 1.19, 2.13), respectively, were more likely to report weighted adequate HL relative to those who were within lower FAI tertile. White collars were more likely to have weighted adequate HL (OR = 1.33, 95%CI = 1.09, 1.61) than blue collars.

**Conclusions:**

Each of education, FAI and occupation was positively associated with health literacy among urban and rural adults in China. The findings have important implications that different SEP indicators can be used to identify vulnerable residents in population-based health literacy promotion campaigns.

## Background

Health literacy (HL) was defined by the World Health Organization (WHO) as the cognitive and social skills which determine the motivation and ability of individuals to gain access to, understand and use information for promoting and maintaining good health [1]. It has been well-documented that low HL was associated with unhealthy lifestyle/behaviors and poor health outcomes in both developing and developed countries [2–7]. Thus, promotion of HL is of public health importance for targeted people to gain/maintain good health. To date, some factors regarding socioeconomic position (SEP), including educational attainment, income, etc., have been examined to be associated with HL among adults in different societies, including China [8–13]. SEP is a useful indicator for identifying sub-populations who are vulnerable to inadequate HL, and subsequently tailored community-based HL intervention strategies can be developed for SEP-specific sub-populations.

SEP is a complex concept, which can be indicated with different measures according to different research context. Each SEP measure usually presents specific information on participant’s socioeconomic position within the society [14]. The most widely used SEP indicators are educational level, occupation and income in public health research area [8]. Of these three SEP indicators, both education and occupation were personally single indicators, while income was mainly used as household-level indicator in previous studies. Recently, a single indicator of SEP regarding income, monthly family average income (FAI), was developed, with consideration of total household income and the number of household members, for population-based studies on association of SEP with diabetes, obesity, stroke in China, showing that it was more realistic and sensitive as a single SEP indicator [15–17].

Although SEP disparities in HL has been identified among adults worldwide, there was no study simultaneously investigate relationship between SEP and HL using FAI, educational attainment and occupation as SEP indicator within a study. Therefore, we used education, occupation and FAI to indicate SEP separately and then to examine the individual association between each of them and HL among urban and rural adults in regional China.

## Methods

### Study design and participants

A large-scale cross-sectional study was conducted among urban and rural adult residents in Nanjing, a mega-city in eastern China, between October and December of 2016. Nanjing had a registered population of approximate 8.3 million within 12 administrative districts in 2016. In China, there is a five-level administration system from the highest to the lowest: Central, Provincial/Municipal, District/County, Street/Town and Neighborhood/Village. In this study, the smallest stratum, Neighborhood/Village, was used as our sampling unit. Household participants were eligible to take part in this study, if they: 1) were local registered residents and had lived in Nanjing for 6+ months prior to the survey; 2) aged 25–69 years old; and 3) had no psychiatric problems/disorders.

### Sample size estimation

The study sample size was estimated based on: 1) available proportion (13.0%) of local residents achieved adequate HL in a pilot survey conducted in 2014 [18]; 2) a multi-stage probability proportionate to size (PPS) sampling approach employed; 3) an expected response rate (90%); and 4) an assumed statistical power of 85%. Thus, approximately 9100 participants could guarantee the sufficient statistical power for stratified analysis in this study.

### Sampling approach

A two-stage PPS sampling approach was applied to select participants from all of 12 administrative districts in Nanjing. Firstly, required numbers of neighborhoods/villages and administrative streets/towns were estimated based on: 1) 72 participants expected to be selected from each neighborhoods/villages, and 2) two neighborhoods/villages chosen from each street/town. Thus, it was estimated that 126 neighborhoods/villages would be chosen from 63 streets/towns in the entire city. Next, the numbers of administrative streets/towns and neighborhoods/villages were computed for and assigned to each district according to the proportion of eligible population (aged 25–69 years old) size within each district to the overall in the whole city. Then, participating administrative streets/towns, neighborhoods/villages and households were selected using random digits. Finally, within each of the selected 72 households within a chosen neighborhoods/village, one subject was randomly recruited from all eligible participants using the KISH Grid sampling method.

Written informed consent was obtained from each participant prior to the interview/survey. This study was approved by Ethics Review Committee of Nanjing municipal Centers for Disease Control and Prevention. All methods were carried out in accordance with relevant guidelines and regulations. And, all personally identifiable information were removed before analysis.

### Instrument and questionnaire

HL was assessed with the Chinese Resident Health Literacy Scale (CRHLS, V2012). The CRHLS was officially developed for health literacy survey among Chinese residents by China National Institute of Health Education in 2012 [19]. The reliability and validity of CRHLS have been examined and reported elsewhere previously [20]. Briefly, a goof reliability of CRHLS was recorded in that the Cronbach’s α coefficient was 0.95 and Spearman-Brown split-half coefficient was 0.94, while its acceptable validity was assessed by confirmatory factor analysis (CFA) and item response theory (IRT) showing that both correlation coefficient and factor loading for each CRHLS item were greater than 0.4 (most of them > 0.5) and the discrimination parameters for all the CRHLS items were between 0.5 and 2.0 (mostly 1.0–2.0) [20]. The CRHLS consists of three domains (knowledge and attitudes, behavior and lifestyle, and health-related skills), including six health-relevant aspects (scientific views of health, infectious diseases, chronic diseases, safety and first aid, medical care, and health-related knowledge).

There were four types of questions in CRHLS: true-or-false, single-answer, multiple-answer and situation questions. A specific score was assigned to each item based on participant’s response to the question. For true-or-false and single-answer questions, “1 point” was assigned to a correct answer, and “0 point” to an incorrect answer. For multiple-answer questions, “2 points” were assigned if the answer was exactly correct, otherwise “0 point” was assigned. For situation questions, participants needed to read a short message and answer single−/multiple-answer questions. “2 points” were assigned for each exactly correct answer to situation question, while “0 point” was recorded if a participant did not answer or answered incorrectly. According to such a scoring rule, the total HL score ranged from 0 to 68 for a participant.

Information on participant’s socio-demographic characteristics and CRHLS was integrated into a big questionnaire, which was self-administered in field survey. In case a participant was not able to complete questionnaire survey by him/herself, research staff were available to provide assistance via face-to-face interview.

### Study variables

#### Outcome variable–HL

The outcome variable was HL, which was treated as a categorical measure in this study. A participant would be regarded as “having adequate HL”, if he/she obtained a score of 54+ (at least 80% of the total score 68), otherwise this person was classified as “not having adequate HL”. The HL level, a population-based term, refers to the proportion of residents with adequate HL in the total study population. Although it was recommended to report weighted HL level, we computed both unweighted and weighted HL level for analysis in this study [21].

#### Explanatory variable–SEP

SEP was the explanatory variable, which was separately indicated with FAI, educational attainment and occupation. FAI was defined as the total monthly incomes of all family members divided by the number of family members (including children and elderly), and was tertiled for the analysis: lower, middle or upper (cut-offs: 1400 and 2800 RMB/month). Educational attainment was classified into three sub-groups according to schooling years completed: 0–9 years, 10–12 years or 13+ years. Occupation was categorized as white-collar or blue-collar based on the work types of participants [22].

#### Co-variables

Some potential confounding factors, including age, gender (men vs. women), residence area (urban vs. rural) and history of selected chronic diseases, were considered as covariates in analysis. Age was categorized as younger (25–39 years), middle (40–54 years) or older (55–69 years). The history of chronic disease referred to the answer to the question “Have you been diagnosed with any of the following chronic diseases by a registered physician?”, and participants were classified into: “Yes” or “No” based on their response to the question.

### Data analysis

All respondents were weighted to the individual’s probability of selection according to the latest census data of Nanjing in 2010. Descriptive analysis was made using chi-square tests (for categorical measures) or t-tests (for continuous variables). Two logistic regression models were introduced to calculate odds ratios (OR) and 95% confidence interval (95%CI) for investigating SEP-HL association. All these three SEP indicators were simultaneously introduced in each model. Model 1 was an unadjusted analysis with SEP indicator as the independent variable. Model 2 was a multivariate analysis with adjustment for age, gender, residence area, history of selected chronic diseases, FAI/education/occupation. Two-sided statistical significance was assessed using *P* < 0.05. Data were double-entered and cleaned with Epi data 3.02, and managed and analyzed using SPSS 21.0 (IBM Corp, Armonk, NY, USA).

## Results

A total of 9168 eligible participants were recruited and 8698 successfully completed the survey, with a response rate of 94.9%. No significant difference was found in age, gender or residential area between our participants and the overall residents aged 25–69 years in this study, showing that the participants were representative of the local overall population. Moreover, there was no significant difference in age, gender or area of residence between respondents and non-respondents in this survey. Table 1 presented participants’ selected characteristics by unweighted and weighted health literacy level in this study. Of those 8698 participants, 71.8% were urban residents; 48.9% were men; 33.2% were participants aged 55–69 years old; 29.5% obtained educational attainment of 13+ schooling years. The unweighted and weighted HL level was 18.0 (95%CI = 17.2, 18.8%) and 19.9% (95%CI = 16.6, 23.6%), respectively, among the overall participants in this study. The HL level significantly differed in categories of residence area, age, FAI, educational attainment, occupation and categories of chronic disease history.

**Table 1:** The unweighted and weighted level of health literacy by selected characteristics of participants in Nanjing, China

Characteristics	Overall participants within each category (n and %)	Participants having adequate HL				
		n	Unweighted		Weighted	
			%(95%*CI*)	*P-*value*	%(95%*CI*)	*P-*value*
Overall	8698 (100)	1566	18.0 (17.2, 18.8)	–	19.9 (16.6, 23.6)	–
Area						
Urban	6241 (71.8)	1334	21.4 (20.4, 22.4)	<0.001	20.8 (17.1, 24.0)	<0.001
Rural	2457 (28.2)	232	9.4 (8.3, 10.6)		11.5 (8.9, 14.7)	
Gender						
Man	4255 (48.9)	729	17.1 (16.0, 18.3)	0.038	19.5 (16.0, 23.6)	0.048
Women	4443 (51.1)	837	18.8 (17.7, 20.0)		20.3 (17.0, 24.1)	
Age (years)						
25–39	2554 (29.4)	701	27.4 (25.7, 29.2)	<0.001	25.1 (21.5, 29.1)	<0.001
40–54	3255 (37.4)	544	16.7 (15.4, 18.0)		18.2 (14.2, 22.9)	
55–69	2889 (33.2)	321	11.1 (10.0, 12.3)		13.9 (10.7, 17.8)	
Education in years						
0–9	3930 (45.2)	344	8.8 (7.9, 9.6)	<0.001	10.2 (7.6, 13.6)	<0.001
10–12	2198 (25.3)	410	18.7 (17.0, 20.3)		18.0 (15.1, 21.3)	
≥ 13	2570 (29.5)	812	31.6 (29.8, 33.4)		28.4 (23.4, 34.1)	
Family average income						
Low	2978 (34.2)	265	8.9 (7.9, 9.9)	<0.001	10.9 (8.3, 14.3)	<0.001
Middle	2478 (28.5)	441	17.8 (16.3, 19.3)		19.0 (15.5, 23.2)	
High	2998 (34.5)	816	27.2 (25.6, 28.8)		25.8 (20.7, 31.7)	
Occupation†						
Blue collar	7426 (85.4)	1170	15.8 (14.9, 16.6)	<0.001	17.9 (14.9, 21.3)	<0.001
White collar	1272 (14.6)	396	31.1 (28.6, 33.7)		29.3 (23.5, 36.0)	
Chronic disease						
No	6472 (74.4)	1269	19.6 (18.6, 20.6)	<0.001	20.9 (17.5, 24.8)	0.003
Yes	2226 (25.6)	297	13.3 (11.9, 14.8)		15.9 (12.3, 20.3)	

* Chi-square was used to make comparisons between subgroups of each variable based on weighted data

† Blue collar = farmer, factory worker, forestry worker, fisher, service stuff, salesperson, house-worker and vehicle driver. White collar = office worker, teacher, doctor, academic researcher and government official

Table 2 showed participants within residence area, gender and age by FAI, education and occupation, separately. The inequality in gender was observed only in educational attainment, while disparities in residence area and age were examined in either of education, FAI or occupation.

**Table 2:** Association of SEP (FAI, education, occupation) and conventional potential confounding factors among urban and rural participants in Nanjing, China

Characteristic	Education in years (n and %*)			*P* **	Family average income (n and %*)			*P* **	Occupation (n and %*)		*P* **
	Lower	Middle	Higher		Lower	Middle	Higher		Blue collar	White collar	
Area											
Urban	1962 (25.6)	1846 (30.0)	2433 (44.4)	< 0.001	1319 (19.5)	2009 (33.1)	2913 (47.4)	< 0.001	5146 (81.5)	1095 (18.5)	< 0.001
Rural	1968 (76.5)	352 (16.2)	137 (7.3)		1724 (68.6)	545 (23.6)	188 (7.8)		2280 (92.4)	177 (7.6)	
Gender											
Male	1809 (27.3)	1121 (28.6)	1325 (44.2)	< 0.001	1479 (23.6)	1221 (31.4)	1555 (45.0)	0.071	3613 (82.2)	642 (17.8)	0.584
Female	2121 (33.9)	1077 (28.8)	1245 (37.4)		1585 (24.3)	1328 (32.2)	1530 (43.6)		3813 (82.8)	630 (17.2)	
Age (years)											
25–39	413 (11.4)	601 (23.52.2)	1540 (66.5)	< 0.001	616 (18.7)	716 (31.8)	1222 (49.5)	< 0.00	2029 (78.1)	525 (21.9)	< 0.001
40–54	1648 (37.3)	860 (31.2)	747 (31.5)		1241 (27.2)	931 (31.2)	1083 (41.6)		2816 (84.4)	439 (15.6)	
55–69	1869 (51.3)	737 (35.5)	283 (13.3)		1205 (28.6)	903 (34.7)	781 (36.8)		2581 (86.7)	308 (13.3)	

* Weighted percentages across row

** Chi-square was used to make comparisons between subgroups of each variable based on weighted data

Table 3 displayed the associations of unweighted and weighted HL level with each of education, FAI and occupation among participants. After adjustment for potential confounding factors, each SEP indicator was in significantly positive relation to both unweighted and weight HL level. Participants who obtained 13+ and 10–12 years education, respectively, had 2.41 (95%CI = 1.60, 3.64) and 1.68 (95%CI = 1.23, 2.29) times odds to record weighted adequate HL compared to their counterparts who were with 0–9 years education. Subjects within upper (OR = 1.92, 95%CI = 1.24, 2.98) and middle FAI tertile (OR = 1.59, 95%CI = 1.19, 2.13), respectively, were more likely to report weighted adequate HL relative to those who were within lower FAI tertile. White collars were also more likely to have weighted adequate HL (OR = 1.33, 95%CI = 1.09, 1.61) than blue collars.

**Table 3:** The unweighted and weighted level of health literacy among residents and their associations with family average income, education and occupation among urban and rural adult participants in Nanjing, China

Characteristics (*N* = 8698)	Number of participants having adequate HL	Unweighted		Weighted	
		% of participants having adequate HL	Adjusted odds ratio* (95%*CI*)	% of participants having adequate HL	Adjusted odds ratio* (95%*CI*)
Overall (all levels)	1566	18.0		19.9	
Education in years					
0–9	344	8.8	1	10.2	1
10–12	410	18.7	1.76 (1.49, 2.09)	18.0	1.68 (1.23, 2.29)
≥ 13	812	31.6	2.48 (2.06, 2.99)	28.4	2.41 (1.60, 3.64)
Family average income					
Low	265	8.9	1	10.9	1
Middle	441	17.8	1.66 (1.39, 1.97)	19.0	1.59 (1.19, 2.13)
High	816	27.2	2.02 (169, 2.41)	25.8	1.92 (1.24, 2.98)
Occupation					
Blue collar	1170	15.8	1	17.9	1
White collar	396	31.1	1.48 (1.28, 1.72)	29.3	1.33 (1.09, 1.61)

* Adjusted odds ratio: with adjustment for age, sex, area of residence, history of chronic disease

Table 4 demonstrated the relationship between SEP indicators and weighted HL level within each stratum by age, gender and residence area. The positive relationship between each SEP indicator and HL was observed among participants living in either urban or rural areas. However, the scenarios of SEP-HL associations were not consistent across strata of participants by gender and age. For men, occupation-HL association was not significant, while FAI-HL association was insignificant for women. Of these three SEP indicators, each of them was in significantly positive relation to HL for residents within younger age-group, while for another two age-groups the scenarios were a little bit complex in that, interestingly, only FAI-HL association was not significant among middle-aged participants but significant among older subjects in this study.

**Table 4:** The effect of SEP on weighted level of HL after stratification by age, gender and area of residence among urban and rural participants in Nanjing, China

Variables	Socioeconomic position category	Level of health literacy (%*)	Adjusted odds ratio** (95% *CI*)
Area			
Urban	Education in years		
	0–9	11.1	1
	10–12	18.0	1.61 (1.15, 2.26)
	≥13	28.3	2.35 (1.53, 3.63)
	Family average income		
	Low	11.9	1
	Middle	19.2	1.53 (1.10, 2.14)
	High	20.9	1.87 (1.17, 3.00)
	Occupation		
	Blue collar	18.8	1
	White collar	29.4	1.30 (1.06, 1.59)
Rural	Education in years		
	0–9	7.7	1
	10–12	17.3	1.61 (1.12, 2.30)
	≥13	38.9	3.21 (1.58, 6.53)
	Family average income		
	Low	8.2	1
	Middle	17.2	1.91 (1.27, 2.87)
	High	23.5	1.95 (1.20, 3.15)
	Occupation		
	Blue collar	10.1	1
	White collar	29.1	1.94 (1.11, 3.38)
Gender			
Male	Education in years		
	0–9	9.1	1
	10–12	17.4	1.84 (1.30, 2.62)
	≥13	27.3	2.73 (1.57, 4.73)
	Family average income		
	Low	8.5	1
	Middle	20.8	2.41 (1.60, 3.64)
	High	24.7	2.47 (1.41, 4.33)
	Occupation		
	Blue collar	18.2	1
	White collar	25.6	1.10 (0.79, 1.53)
Female	Education in years		
	0–9	11.2	1
	10–12	18.5	1.54 (1.09, 2.18)
	≥13	30.0	2.17 (1.46, 3.21)
	Family average income		
	Low	13.4	1
	Middle	17.2	1.10 (0.75, 1.61)
	High	27.2	1.58 (0.99, 2.48)
	Occupation		
	Blue collar	17.6	1
	White collar	33.6	1.59 (1.21, 2.10)
Age (years)			
25–39	Education in years		
	0–9	15.1	1
	10–12	22.8	1.61 (1.03, 2.52)
	≥13	27.6	1.72 (1.12, 2.64)
	Family average income		
	Low	14.2	1
	Middle	26.0	2.05 (1.33, 3.16)
	High	29.2	2.31 (1.48, 3.62)
	Occupation		
	Blue collar	23.4	1
	White collar	31.3	1.30 (1.02, 1.65)
40–54	Education in years		
	0–9	8.7	1
	10–12	15.6	1.75 (1.10, 2.78)
	≥13	31.9	3.84 (2.24, 6.57)
	Family average income		
	Low	10.6	1
	Middle	16.0	1.25 (0.86, 1.80)
	High	24.8	1.47 (0.85, 2.52)
	Occupation		
	Blue collar	15.6	1
	White collar	32.1	1.44 (1.03, 2.01)
55–69	Education in years		
	0–9	10.3	1
	10–12	16.3	1.40 (0.83, 2.37)
	≥13	21.0	1.64 (0.86, 3.14)
	Family average income		
	Low	7.6	1
	Middle	12.8	1.42 (0.95, 2.12)
	High	20.1	2.16 (1.13, 4.15)
	Occupation		
	Blue collar	13.2	1
	White collar	18.3	0.99 (0.60, 1.65)

* Weighted level of health literacy

** Odds ratio with adjustment for age, sex, area of residence, existence or not of chronic disease based on weighted data

## Discussion

We performed this population-based study for better understanding HL disparities in SEP indicated, separately, with education, FAI and occupation among urban and rural adults in regional China. The findings were interesting and important: 1) each of education, FAI and occupation was significantly associated with HL among either urban or rural adult residents; 2) such a significant association was not observed consistently among gender−/age-specific sub-groups. It suggested that each of education, FAI and occupation could be used as indicator of SEP for examining SEP disparities in HL, but each SEP indicator might have different and specific performance on identifying HL-vulnerable sub-populations.

Health literacy, as cognitive knowledge and social skills, may change for residents in a society with economic development and social norm transition, particularly in developing communities [1]. Even for the same individual, he/she may have different HL levels at different time points. Consequently, the association between SEP and HL may also change in a society with time going on. So it is of importance to investigate SEP-HL relationship dynamically/ periodically in a long term for the purpose of tailored population-level HL intervention, even if there are similar documents previously available in the same country or different societies.

Education, occupation and income were widely used to measure SEP for exploring SEP-HL relationship worldwide [8–13]. Of these three SEP indicators, education was consistently measured with participant’s schooling years completed, and occupation was usually categorized as white or blue collars [21]. However, as for income, it was usually used as a household-level indicator of SEP, e.g., total income per family and household-level deprivation score [23], without consideration of the number of household members, which could not reflect the realistic SEP for household members individually. Obviously, the same amount of total household income could not have the same meaning for families with different members. Given the same amount of household income, the more family members, the less average amount of money available for each member. In our study, we used family average income as an index to measure participants’ SEP, not only because family size was involved in consideration, but also because it has been demonstrated to be a more realistic indicator than household-level income in public health research on SEP and chronic diseases [15–17]. In China, most families have only one child, but meanwhile a lot of families have their parents and/or grandparents living together, especially in the rural areas. For those elders, they may have lower retirement salaries or even no incomes (rural elders) at all. Thus, it is of particular meaningfulness to use FAI other than household-level income to predict SEP for investigating SEP-disparities in public health issues in China.

Instruments used for assessing HL varied across studies worldwide, as different scales were designed aiming to measure specific aspects of health literacy [24]. Thus, it is difficult to make direct comparison of HL level between studies using different HL instruments. Although different HL assessment instruments were used, our findings regarding relationship between education, income and HL were in line with those reported from other communities in the world [9–13, 25, 26]. Possible mechanisms could be used to explain relationship between education, income and HL. With regard to education, it has been identified that educational attainment could help participants not only obtain knowledge and skills, but also translate them into better understanding of health literacy [27, 28]. This might, at least in part, explain the positive education-HL association in this study. As for income, it has also been documented that a resident with high income was consistently more likely to access to health information, to obtain health-care resources and to receive the services [12, 29–31]. Thus, this might also partially underlie the positive relationship between FAI and HL in our study.

The scenario of occupation-HL association was a little bit complex, because the definitions and classifications of occupation were different in previous studies. For example, Michou et al classified occupation into three categories: skilled, semi-skilled and unskilled, and reported no association between occupation and HL [11]; Jeong et al categorized occupation as employed and unemployed, and found no significant occupation-HL association [32]. However, Joveini et al grouped participants into six occupation categories in their study, and documented that civil servant, student or self-employed individuals were more likely to obtain adequate HL than unemployed people [33]. And, Wu et al also reported technical/professional workers were more likely to possess adequate HL than manual workers in Chinese population [12].

In our study, occupation was categorized as the white collar or the blue collar based on the work types of participants [21]. It was observed that white collars were more likely to have high HL level than blue collars. This might be explained by that, compared to blue collars, white collars might care more about their health condition and have more opportunities to access to social resources and healthcare-related information, consequently helping them improve HL [34]. Our finding on the occupation-HL relationship was in line with some previous studies [35, 36], but inconsistent with others [11, 32]. This might be due to the different definition and classification of occupation between our study and others.

This study had several strengths. First, data were gathered from urban and rural general population of men and women with a very high response rate. Second, education, FAI and occupation were used as individual indicator of SEP to explore their associations with HL in a population-based study. This study is the first one to investigate SEP-HL association using education, FAI and occupation to indicate SEP in China. Third, interesting findings were observed in that either education, FAI or occupation was positively associated with HL among adult residents in China. This suggested that each of these three indicators could be used to identify SEP-vulnerable sub-population for community-level HL intervention. So, different SEP indicators should be encouraged to be applied for investigating SEP-HL association at population level.

Despite these strengths, some limitations of this study deserve mention. First, this study was a cross-sectional survey, which could not allow us to infer causality for the SEP–HL relationship. Second, the family income was self-reported, which implied potential recall bias. In future, longitudinal studies are needed to investigate how change in socioeconomic position will exert impact on the change in health literacy. Third, the instrument used to measure HL in our study (CRHLS, V2012) might have a little bit different health-related knowledge points from other HL instruments used in different societies, although it was developed and validated specifically for Chinese people [19, 20]. Some knowledge and practices included in a HL instrument may have different ideas for people who have different cultural tradition and/or social norms worldwide. Therefore, HL instrument shall be culture-specific/sensitive, and then our HL instrument was not only acceptable but also “shall-do” in a study for assessing HL among Chinese people. Considering that China has a population of more than 1.4 billion, it is very important and wonderful that an instrument can be used to measure HL for such a huge number of people.

## Conclusions

As SEP indicator, each of education, family average income and occupation was positively associated with health literacy among men and women in urban and rural areas in China. The findings have important public health implications that different SEP indicators can be used to identify sensitive residents in population-based health literacy promotion campaigns.
